# Electronic band-gap modified passive silicon optical modulator at telecommunications wavelengths

**DOI:** 10.1038/srep16588

**Published:** 2015-11-13

**Authors:** Rui Zhang, Haohai Yu, Huaijin Zhang, Xiangdong Liu, Qingming Lu, Jiyang Wang

**Affiliations:** 1 State Key Laboratory of Crystal Materials and Institute of Crystal Materials, Shandong University, Jinan 250100, China; 2 School of Physics, Shandong University, Jinan 250100, China; 3 School of Chemistry and Chemical Engineering, Shandong University, Jinan 250100, China

## Abstract

The silicon optical modulator is considered to be the workhorse of a revolution in communications. In recent years, the capabilities of externally driven active silicon optical modulators have dramatically improved. Self-driven passive modulators, especially passive silicon modulators, possess advantages in compactness, integration, low-cost, etc. Constrained by a large indirect band-gap and sensitivity-related loss, the passive silicon optical modulator is scarce and has been not advancing, especially at telecommunications wavelengths. Here, a passive silicon optical modulator is fabricated by introducing an impurity band in the electronic band-gap, and its nonlinear optics and applications in the telecommunications-wavelength lasers are investigated. The saturable absorption properties at the wavelength of 1.55 μm was measured and indicates that the sample is quite sensitive to light intensity and has negligible absorption loss. With a passive silicon modulator, pulsed lasers were constructed at wavelengths at 1.34 and 1.42 μm. It is concluded that the sensitive self-driven passive silicon optical modulator is a viable candidate for photonics applications out to 2.5 μm.

Silicon is the basis of microelectronics in the information age and its dominance likely will continue in the foreseeable future. Silicon photonics devices provide a platform for integrating all optical components onto a single chip and improving the performance of systems in many applied areas^1^,^2^,^3^,^4^,^5^,^6^. In photonics, gains for light emission, nonlinear optical devices for frequency-shifting and optical modulators for controlling operations are the fundamental functions. However, the structure and composition of a material determine its properties and applications, and so the two main challenges in the development of silicon photonics are the indirect band-gap and centro-symmetry in the structure of intrinsic silicon, which impedes lasing and eliminates second-order nonlinear optical functions such as frequency-doubling and electro-optical modulation^5^,^7^. By modulating the microstructure of silicon^8^,^9^ or doping it with active rare-earth ions^6^, silicon-based light-emitting devices have been developed. By breaking the crystal symmetry of silicon^2^,^10^, second harmonic generation and sum frequency generation have been realized. Third-order nonlinear optical properties is independent of the centro-symmetry in the structure, and by using the greater third-order nonlinear properties of silicon, stimulated Raman scattering and third-harmonic generation have become promising pathways to produce silicon lasers^2^,^3^,^4^,^5^,^11^.

In the case of silicon optical modulators, active silicon electro-optical modulators that depend on second-order optical nonlinearity have been developed and are considered to revolutionary short-reach connectivity, for both conventional data networks and intra-/interchip data links^12^,^13^,^14^,^15^. Optical modulators include both active and passive types^16^. Active optical modulators, e.g. silicon electro-optical modulators must be externally driven. Passive optical modulators are based on the nonlinear reaction of the material to light and have advantages in compactness, integration, low-cost, etc. The Kerr effect and saturable absorption are the main phenomena used in passive optical modulators, both of which represent third-order optical nonlinearities, where the former is determined by the real part of the optical susceptibility and the latter is related to the imaginary part^10^. Compared with the Kerr effect, saturable absorption is a modulation of the absorption and has advantages in some aspects, such as tunable and large modulation depth. However, besides the loss generated during saturable absorption process which is determined by the sensitivity of the absorption to light intensity, the 1.12 eV indirect band-gap of silicon constrains its application in passive modulators, especially at telecommunications wavelengths e.g. 1.3 (0.95 eV) and 1.5 μm (0.8 eV)^7^. Modifying the electronic band structure of silicon represents the challenge in the development of passive silicon modulators^7^,^10^. Recently, theoretical calculations indicated that a dopant-induced impurity electronic band located within the silicon band-gap could be generated and controlled by supersaturated doping with chalcogen elements^17^. The linear infrared photoresponse of supersaturated implanted silicon was experimentally investigated and showed much stronger absorption than intrinsic germanium^18^,^19^. Here, we address the third-order optical nonlinear response, saturable absorption, of crystalline silicon supersaturated doped with sulfur and its application as a passive optical modulator at telecommunications wavelengths. Exploiting the unique nonlinear optical response of sulfur-doped silicon, we have found that it possesses quite sensitive nonlinear saturable absorption and broadband response out to 2.5 μm. Moreover, modulated pulses at the telecommunications wavelengths were also produced using the fabricated silicon sample as the optical modulator.

## Results and Discussion

**Figure 1:**
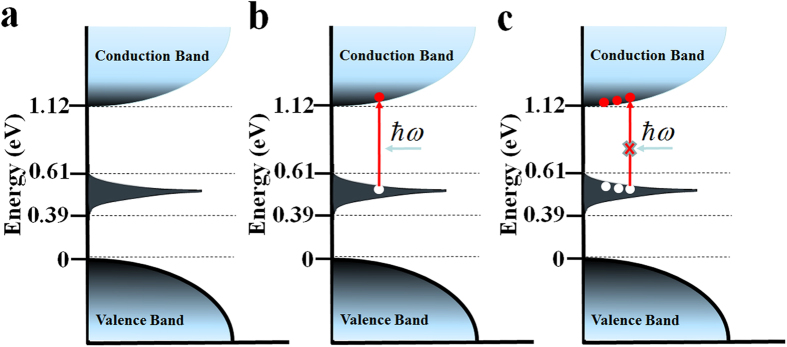
Absorption of light in sulfur-doped silicon. (**a**) Band-gap structure of sulfur-doped silicon. The band between conduction and valence bands represents the impurity band generated by implantation of sulfur ions. (**b**) Schematic excitation process responsible for absorption of light in sulfur-doped silicon. The red arrow indicates optical interband transition from impurity to conduction band. (**c**) Under irradiation by high-intensity light, photogenerated carriers cause states in conduction and valence bands to fill, blocking further absorption and producing the saturable absorption.

The silicon crystal sample was cut along the <100> direction with a thickness of 381 μm and polished on opposite faces perpendicular to this direction. The ^32^S^+^ ion was implanted into the silicon crystal at 95 keV to a dose of 3 × 10^15^ cm^−2^ at room temperature. Based on theoretical calculation^17^, it is assumed that sulfur is substitutional in the silicon crystal since the formation energy of the substitutional positioning process is 2.84 eV per sulfur ion, which is lower than interstitial implantation. An intermediate band generated by the sulfur impurities appears, as shown in Fig. 1a, with a bandwidth of 0.22 eV, 0.39 eV above the valence band maximum and 0.51 eV below the conduction band minimum. The impurity band is fully occupied and the transfer of electrons from the impurity to the conduction band is dominant for absorption in the telecommunication wavelength band with an absorption peak at 0.8 eV corresponding to the wavelength of 1.54 μm^17^,^18^. Therefore, we can assume that with irradiation under light with photon energy greater than 0.51 eV, electrons in the impurity band are transferred to the conduction band with the absorption of the light.

**Figure 2:**
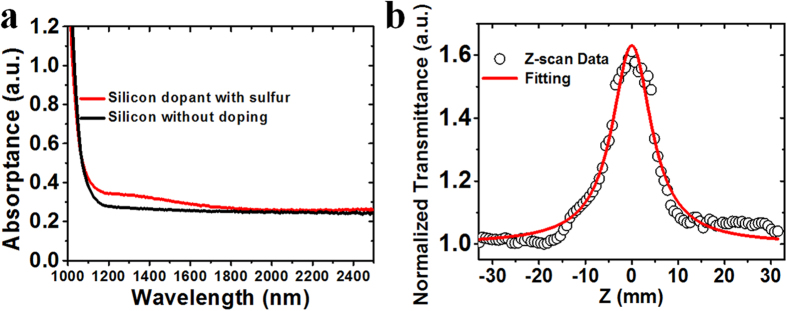
Absorption properties of sulfur-doped silicon. (**a**) Absorption spectra of sulfur-doped silicon and silicon single crystal samples measured with spectrophotometer. (**b**) Nonlinear absorption of sulfur-doped silicon at 1.55 μm achieved by the Z-scan technique.

In order to verify that electron transfer from the impurity to the conduction band occurs, the absorption spectrum of the silicon samples was measured with a V-570 JASCO UV/VIS/NIR spectrophotometer, and the results are shown in Fig. 2a. For comparison, the spectrum of an undoped silicon sample is also presented in this figure. From the figure, it is seen that the difference between the absorption bands of sulfur doped silicon and undoped silicon samples is indistinguishable in wavelength range from 2.0 to about 2.5 μm corresponding to 0.51 to 0.62 eV jump from the impurity to the conduction band, as shown in Fig. 1a in agreement with theoretical calculations and previous experimental results^17^,^19^. The absorption band covers the telecommunications wavelengths of 1.3 and 1.5 μm. From the band-gap structure and absorption spectrum shown in Fig. 1, it is seen that when the sulfur-doped silicon sample is irradiated by light with photon energy greater than 0.51 eV, electrons are transferred from the impurity band to the conduction band. It can also be theoretically predicted that under strong excitation, photogenerated carriers fill the states in the conduction and impurity bands, blocking further absorption and thus producing saturable absorption, as shown in Fig. 1b,c. The result represents a third order nonlinear response of the silicon sample to incident light, and allows the sample to be used as an optical modulator.

In order to investigate the saturable absorption properties of the sulfur-doped silicon sample, the Z-scan technique was applied. A mode-locked fiber laser with a wavelength of 1.55 μm was used as the pump source. The fiber laser has a pulse duration and repetition rate of 565 fs and 20.13 kHz, respectively. Through a focusing lens, the minimum focus point had a radius of 30 μm. The Z-scan experiment data of the sulfur-doped silicon sample are shown in Fig. 2b. According to the theoretical calculation^20^, the Z-scan data was fitted with the following equation:


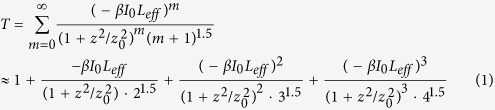


where *T* is the normalized transmittance, *β* is nonlinear absorption coefficient, *I*_0_ is peak intensity at the focus point *z* = 0, 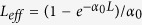 is the effective length, *L* is the length of sample, *α*_0_ is linear absorption coefficient, *z* is the position of the sample and *z*_0_ is the Rayleigh length. The fitting result shows that the nonlinear absorption coefficient *β* of the sample is determined to be −0.834 cm/MW. The saturable intensity, the irradiation intensity reducing the linear absorption coefficient to be 0.5α_0_, of the sample is calculated to be 3.19 MW/cm^2^, which is larger than graphene (0.71 to 0.61 MW/cm^2^) but about three orders smaller than MoS_2_ (2.45 GW/cm^2^ or 413 GW/cm^2^)^21^,^22^,^23^,^24^, which indicates that the prepared sample has strong three-order nonlinear response and large three-order nonlinear absorption coefficient inversely proportional to the saturation intensity, is a material that is considered to be a promising optical modulator at telecommunications wavelengths. The much smaller saturation intensity also indicates that the sulfur-doped silicon sample is easily saturate, and is quite sensitive to incident light. It can be predicted that the absorption loss for bleaching the sample is much small, especially for short-pulse and high peak power lasers at telecommunications wavelengths, and as a result, the fabricated sample should be a promising loss-negligible optical modulator, especially at telecommunications wavelengths.

In order to exclude the burnt effect of the sample in the Z-scan experimental process, the experiments about the laser-induced damage threshold has been implemented. A mode-locked fiber laser with a wavelength of 1.55 μm was used as the source. The laser has a pulse width and repetition of 602 fs and 21 MHz, respectively. With a focus system, the laser beam was focused to be 13 μm in the radius. The silicon sample was put at the focus of the beam. However, no damage was observed in the sample at the peak intensity of 2.24 GW/cm^2^ much larger than maximum incident intensity 43.9 MW/cm^2^ used in the Z-scan experiments. The result indicates that prepared sample has a laser-induced damage threshold larger than 2.24 GW/cm^2^ and nonlinear response measured in Z-scan experiments was generated by the saturable absorption.

**Figure 3:**
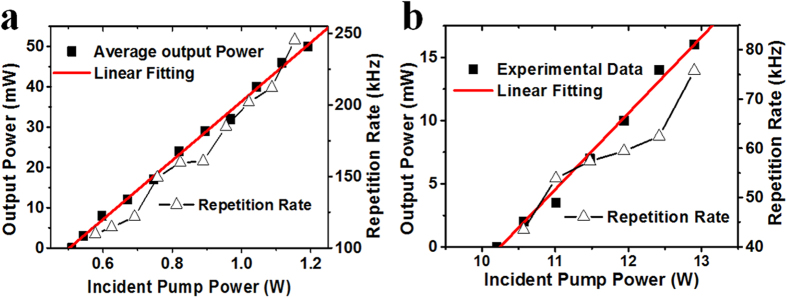
Average output power and repetition rate of passively modulated pulsed laser. (**a**) Pulsed Nd:GdVO_4_ laser performance at the wavelength of 1.34 μm with maximum average output power of 50 mW and repetition rate of 245.5 kHz. (**b**) Pulsed Nd:YGG laser performance at the wavelength of 1.42 μm with maximum output power of 16 mW and repetition rate of 75.76 kHz.

**Figure 4:**
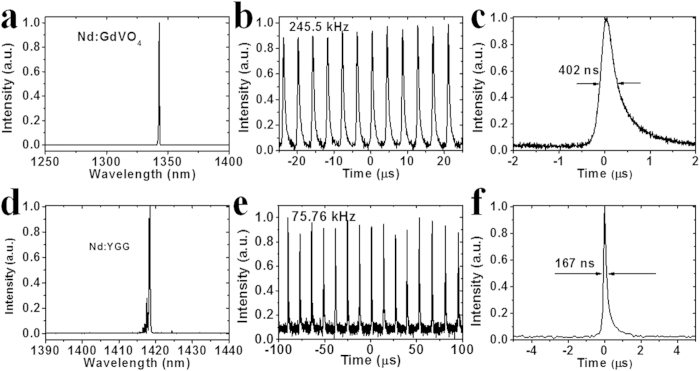
Pulsed laser spectra and pulses modulated by sulfur-doped silicon. (**a**) Pulsed Nd:GdVO_4_ laser spectrum at center wavelength of 1.34 μm. (**b**) Pulse trains of Nd:GdVO_4_ laser at wavelength of 1.34 μm with repetition rate of 245.5 kHz. (**c**) Single pulse profile of Nd:GdVO_4_ laser at wavelength of 1.34 μm with pulse width of 402 ns. (**d**) Pulsed Nd:YGG laser spectrum at center wavelength of 1.42 μm. (**e**) Pulse trains of Nd:YGG laser at wavelength of 1.42 μm with repetition rate of 75.76 kHz. (**f**) Single pulse profile of Nd:YGG laser at r wavelength of 1.42 μm with pulse width of 167 ns.

To investigate the optical modulation properties of the sulfur-doped silicon sample, it was used as a pulse modulator in two solid-state lasers operating at wavelengths of 1.34 and 1.42 μm employing Nd:GdVO_4_ and Nd:Y_3_Ga_5_O_12_ (Nd:YGG) crystals, respectively. A detailed description of the experimental configuration is shown in the *Method section*. With a digital oscilloscope, the pulse width and repetition rate of the pulsed lasers modulated by the prepared silicon sample were recorded. The average output power and repetition rate are shown in Fig. 3a,b at wavelengths of 1.34 and 1.42 μm, respectively. From the output power, repetition rate and pulse width measurement, the pulse energy and peak power of the lasers was determined. It was found that the average output power and repetition rate for both lasers increased with incident pump power, in good agreement with Q-switching theory under modulation by a passive optical modulator^25^,^26^. The maximum output power and repetition rate are 50 mW and 245.5 kHz, respectively, at an operating wavelength of 1.34 μm, with maximum output energy of 0.21 μJ. The pulse width of the Nd:GdVO_4_ pulsed laser decreased from 681 to 402 ns with increase in incident pump power. Figure 4a–c present the operating wavelength spectrum, pulse train with a repetition rate of 245.5 kHz, and single pulse profile with a pulse width of 402 ns of the passively modulated Nd:GdVO_4_ laser, respectively. In the Nd:YGG laser operating at 1.42 μm, the maximum output power is 16 mW with a maximum repetition rate of 75.76 kHz with a minimum pulse width of 167 ns and a maximum pulse energy of 0.22 μJ. The laser wavelength spectrum, pulse train with repetition rate of 75.76 kHz and a single pulse profile with pulse width of 167 ns are shown in Fig. 4e–g, respectively. All the laser experiments demonstrated that the sulfur-doped silicon sample exhibits saturable absorption properties and can be applied as a broadband passive modulator at telecommunications wavelengths. Combining the ease of integration and low cost of silicon crystals, it is concluded that the prepared silicon sample has promising applications as an optical modulator.

In conclusion, with the aim of constructing a loss-negligible passive optical modulator for application at telecommunications wavelengths, a silicon optical modulator was fabricated. By implanting sulfur ions in the silicon crystal, an impurity band was generated which is located between the valence and conduction bands, with a gap in the range of 0.51 ~ 0.62 eV between impurity and conduction bands. Under irradiation by high-intensity light, the third-order nonlinear optical response, saturable absorption, was generated by photogenerated carriers that move between the impurity and conduction bands. Saturable absorption of the prepared samples at 1.5 μm was demonstrated and shows that the sample has a strong third-order nonlinear response to light. Passive pulsed lasers at 1.3 and 1.4 μm were produced in standard solid-state lasers modified with the prepared silicon sample as a passive pulse modulator. All the results show that the sulfur-doped silicon sample holds promise as a pulse modulator with negligible loss and may play a valuable role in enhancing connectivity especially in computing fields at telecommunications wavelengths band^15^,^26^,^27^.

## Methods

### Lasers configuration with silicon optical modulator

For the pulsed laser experiments modulated by the sulfur-doped silicon sample, we used a two-mirror resonator. The detailed description of the experimental configuration is shown in the Supplementary Information. The pump source was a fiber-coupled diode at an emission wavelength of 808 nm, with a numerical aperture of 0.22 and a 400 μm core diameter. The prepared silicon sample was inserted into the resonator between the gain material and output coupler. With a focusing system, pump power was focused through the front input mirror, antireflection (AR) coated at the pump wavelength and highly reflective (HR) at the laser wavelength into the laser crystal. The laser transmission surfaces of all the crystals were polished and uncoated. All laser crystals were mounted in Cu holders with cooling water operating at 15 °C.

In the 1.3 μm laser, a Nd:GdVO_4_ crystal with the dimensions of 3 mm × 3 mm × 10 mm (b × c × a) was used as the gain material. The doped Nd^3+^ ion concentration was 0.5 at%. The front mirror was concave with a 200 mm radius of curvature and AR coated at 1.06 μm. The output coupler was a plane mirror AR coated at 1.06 μm with a transmission of 5% at 1.3 μm. The AR coating is intended to inhibit oscillation at 1.06 μm. For the 1.42 μm laser, a Nd:YGG crystal with dimensions of 2 mm × 2 mm × 6 mm cut along the <111> direction was used as the gain material. The doped Nd^3+^ concentration in the YGG crystal was 1 at%. In the cavity, both the front and output mirrors were AR coated at 1.06 μm and 1.3 μm in order to inhibit oscillation at these two wavelengths. The front mirror was planar and the output mirror had a radius of curvature of 50 mm with a transmission of 5% at 1.42 μm. The length between the front and output mirrors was 45 mm.

## Additional Information

**How to cite this article**: Zhang, R. *et al*. Electronic band-gap modified passive silicon optical modulator at telecommunications wavelengths. *Sci. Rep.* **5**, 16588; doi: 10.1038/srep16588 (2015).

## Supplementary Material

Supplementary Information

## References

[b1] Paul D. J. Silicon photonics: a bright future? Electron Lett. 45, 582–584 (2009).

[b2] Leuthold J., Koos C. & Freude, W. Nonlinear silicon photonics. Nat. Photon. 4, 535–544 (2010).

[b3] Healy N. . Extreme electronic bandgap modification in laser-crystallized silicon optical fibres. Nat. Mater. 13, 1122–1127 (2014). 25262096 10.1038/nmat4098

[b4] Canham L. T. Silicon quantum wire array fabrication by electrochemical and chemical dissolution of wafers. Appl. Phys. Lett. 57, 1046–1038 (1990).

[b5] Walters R. J., Kalkman J., Polman A., Atwater H. A. & de Dood M. J. A. Photoluminescence quantum efficiency of dense silicon nanocrystal ensembles in SiO_2_. Phys. Rev. B 73, 132202 (2006).

[b6] Fujii M., Yoshida M., Kanzawa Y., Hayashi S. & Yamamoto K. 1.54 μm photoluminescence of Er^3+^doped into SiO_2_ films containing Si nanocrystals: Evidence for energy transfer from Si nanocrystals to Er^3+^. Appl. Phys. Lett. 71, 1198–1200 (1997).

[b7] Avrutsky I. & Soref R. Phase-matched sum frequency generation in strained silicon waveguides using their second-order nonlinear optical susceptibility. Opt. Express. 19, 21707–21716 (2011). 22109021 10.1364/OE.19.021707

[b8] Hon N. K., Tsia K. K., Solli D. R. & Jalali B. Periodically poled silicon. Appl. Phys. Lett. 94, 091116 (2009).

[b9] Takahashi Y. . A micrometre-scale Raman silicon laser with a microwatt threshold. Nat. 498, 470–474 (2013). 10.1038/nature12237 23803846

[b10] Corcoran B. . Green light emission in silicon through slow-light enhanced third-harmonic generation in photoniccrystal waveguides. Nat. Photon. 3, 206–210 (2009).

[b11] Rong H. . A continuous-wave Raman silicon laser. Nat. 433, 725–728 (2005). 10.1038/nature03346 15716948

[b12] Jacobsen R. S. . Strained silicon as a new electro-optic material. Nat. 441, 199–202 (2006). 10.1038/nature04706 16688172

[b13] Hochberg M. . Towards a millivolt optical modulator with nano-slot waveguides. Opt. Express 15, 8401–8410 (2007). 19547171 10.1364/oe.15.008401

[b14] Baehr-Jones T. . Nonlinear polymer-clad silicon slot waveguide modulator with a half wave voltage of 0.25 V. Appl. Phys. Lett. 92, 163303 (2008).

[b15] Chmielak B. . Pockels effect based fully integrated, strained silicon electro-optic modulator. Opt. Express 39, 17212–17219 (2011). 21935084 10.1364/OE.19.017212

[b16] Reed G. T., Mashanovich G., Gardes F. Y. & Thomson D. J. Silicon optical modulators. Nat. Photon. 4, 518–526 (2010).

[b17] Sánchez K., Aguilera I., Palacios P. & Wahnón P. Formation of a reliable intermediate band in Si heavily coimplanted with chalcogens (S, Se, Te) and group III elements (B, Al). Phys. Rev. B. 82, 165201 (2010).

[b18] Simmons C. B. . Enhancing the infrared photoresponse of silicon by controlling the Fermi level location within an impurity band. Adv. Funct. Mater. 24, 2852–2858 (2014).

[b19] Bob B. P. . Fabrication and subband gap optical properties of silicon supersaturated with chalcogens by ion implantation and pulsed laser melting. J. Appl. Phys. 107, 123506 (2010).

[b20] Sheik-Bahae M., Said A., Wei T.-H., Hagan D. J. & Van Stryland E. W. Sensitive measurement of optical nonlinearities using a single beam. IEEE J. Quantum Electron. 26, 760–769 (1990).

[b21] Wang S. . Broadband Few‐Layer MoS_2_ Saturable Absorbers. Adv. Mater. 26, 3538–3544 (2014). 24700738 10.1002/adma.201306322

[b22] Wang K. P. . Ultrafast Saturable Absorption of Two-Dimensional MoS_2_ Nanosheets. Acs Nano 7, 9260–9267 (2013). 24090402 10.1021/nn403886t

[b23] Bao Q. L. . Atomic-Layer Graphene as a saturable absorber for ultrafast pulsed lasers. Adv. Funct. Mater. 19, 3077–3083 (2009).

[b24] Sun Z. . Graphene mode-locked ultrafast laser. ACS Nano. 4, 803–810 (2010). 20099874 10.1021/nn901703e

[b25] Zhang X. Y. . Optimization of Cr^4+^-doped saturable-absorber Q-switched lasers. IEEE J. Quantum Electron. 33, 2286–2294 (1997).

[b26] Degnan J. J. Optimization of passively Q-switched lasers. IEEE J. Quantum Electron. 31, 1890–1901 (1995).

[b27] Dejonckheere A. . All-optical reservoir computer based on saturation of absorption. Opt. Express 22, 10868–10881 (2014). 24921786 10.1364/OE.22.010868

