# Supplementary material for: Electronic band-gap modified passive silicon optical modulator at telecommunications wavelengths
Source: Sci Rep. 2015 Nov 13;5:16588. doi: 10.1038/srep16588 (PMC4643244; doi:10.1038/srep16588)
Supplement: Supplementary Information [file srep16588-s1.doc]

**Supplementary Information**

Electronic band-gap modified passive silicon optical modulator at telecommunications wavelengths

Rui Zhang,^1^ Haohai Yu,^1^* Huaijin Zhang,^1^** Xiangdong Liu ^2^ Qingming Lu^3^ and Jiyang Wang^1^

^1^State Key Laboratory of Crystal Materials and Institute of Crystal Materials, Shandong University, Jinan 250100, China

^2^ School of Physics, Shandong University, Jinan 250100, China

^3^ School of Chemistry and chemical engineering, Shandong University, Jinan 250100, China

* and **Corresponding authors: Haohai Yu, and Huaijin Zhang, respectively

Address: State Key Laboratory of Crystal Materials and Institute of Crystal Materials, Shandong University, Jinan 250100, China

Fax: +86-531-88574135

*Email: haohaiyu@sdu.edu.cn

**Email: [huaijinzhang@sdu.edu.cn](mailto:huaijinzhang@sdu.edu.cn)

*Lasers configuration with silicon optical modulator*: For the pulsed laser experiments modulated by the silicon sample dopant with sulfur ions, we used a two-mirror resonator as shown in Fig. S1. The pump source was a fiber-coupled diode at an emission wavelength of 808 nm, with a numerical aperture of 0.22 and a 400 μm core diameter. The prepared silicon sample was inserted into the resonator between the gain material and output coupler (OC). With a focusing system, pump power was focused through the front input mirror M1, antireflection (AR) coated for the pump wavelength and highly reflective (HR) for the laser wavelength into the laser crystals. The laser transmission surfaces of all the crystals were polished and uncoated. All the laser crystals were mounted in Cu holders with cooling water operating at 15 °C. The output laser was separated by a splitter M2. The average output power was recorded by a power meter and the pulse trains were monitored by a digital oscilloscope.

In the 1.3 µm laser, a Nd:GdVO_4_ crystal was used as the gain material with the dimensions of 3 mm × 3 mm × 10 mm ( b × c × a ) The Nd^3+^ ions doping concentration is 0.5 at%. The front mirror M1 was concave with a 200 mm radius with AR coated at 1.06 μm and the OC was a plane mirror AR coated at 1.06 μm with the transmission of 5% at 1.3 μm. The AR coating is for inhibiting oscillation at 1.06 μm. For the 1.42 μm laser, a Nd:YGG crystal used for the 1.42 μm laser gain had dimensions of 2 mm × 2 mm × 6 mm cut along the <111> direction. The Nd^3+^ doping concentration in the YGG crystal was 1 at%. In the cavity, both the front and output mirrors were AR coated at 1.06 μm and 1.3 μm for inhibiting oscillation at these two wavelengths. The front mirror M1 was plane and the OC had a radius of curvature of 50 mm with a transmission of 5% at 1.42 μm. The length between the front and output mirrors was 45 mm.


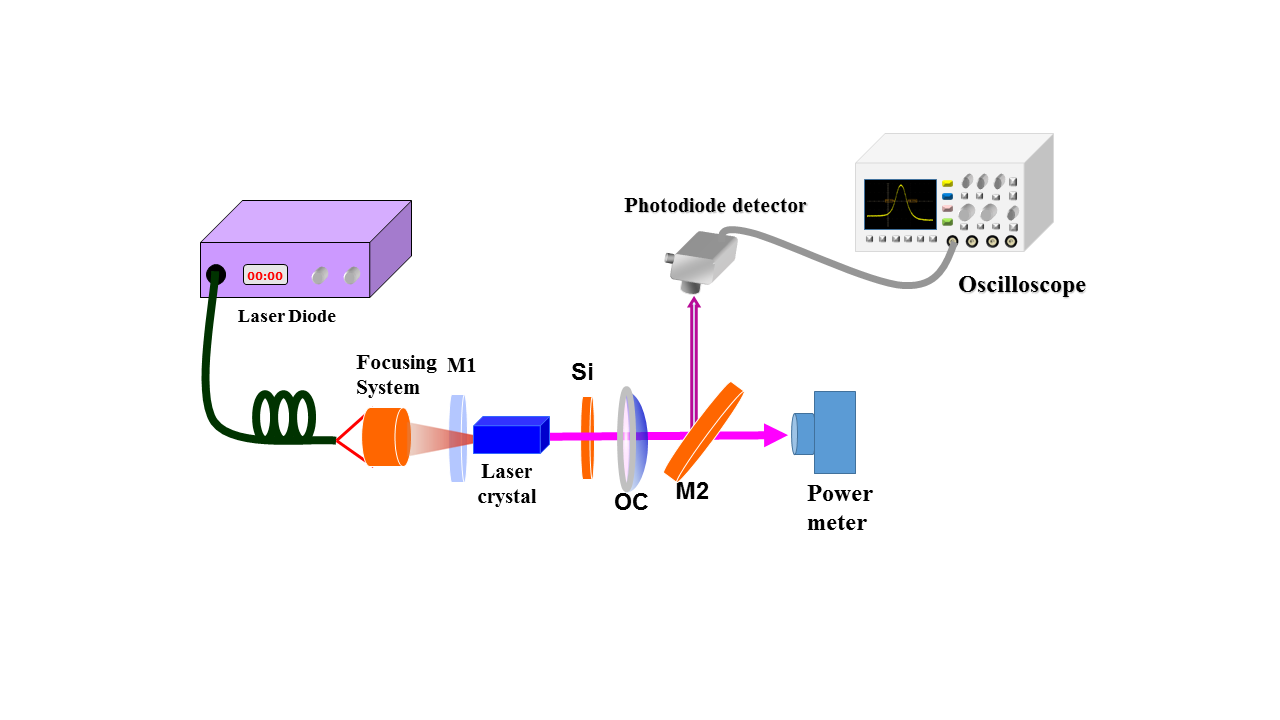


Figure S1.The configuration of pulsed lasers with silicon as the optical modulator
